# Efficacy of a mobile-based approach-avoidance task training (PROTECTapp) for problematic usage of the internet in young adults: A randomized controlled trial

**DOI:** 10.1556/2006.2025.00551

**Published:** 2026-04-07

**Authors:** Daniel Huth, Sebastian Brand, Katajun Lindenberg

**Affiliations:** Department of Clinical Psychology of Childhood and Adolescence, Heidelberg University, Heidelberg, Germany

**Keywords:** problematic internet use, mobile-based intervention, approach bias modification, university students, randomized controlled trial, efficacy

## Abstract

**Background and aims:**

Problematic usage of the internet (PUI) has been linked to impaired mental health and academic functioning in young adults. This randomized controlled trial evaluated the efficacy of a 3-week mobile-based approach–avoidance task (AAT) training (PROTECTapp) for reducing PUI in university students.

**Methods:**

Ninety-two participants (*M*_age_ = 22.00 years, 69.6% women) with elevated levels of PUI were randomized to the PROTECTapp intervention (*n* = 45) or a waitlist control group (*n* = 47). Primary outcomes were PUI severity and internet-related craving. Secondary outcomes included motivation to change, psychopathological symptoms and academic functioning. Participants were assessed at baseline and postintervention; the intervention group completed additional 3- and 12-week follow-ups.

**Results:**

Intention-to-treat analyses indicated greater reductions in PUI following the PROTECTapp intervention compared to the waitlist (*p* = .003; *d* = −0.80, 95% CI [−1.23, −0.38]). No significant effects emerged for craving or broader psychological outcomes (*p*s > .05), though favorable effects were observed on motivation to change (ambivalence: *p* = .020; *d* = −0.24, 95% CI [−0.65, 0.17]; taking steps: *p* = .002; *d* = 0.45 [0.04, 0.87]). Satisfaction with the intervention was moderate (*M* = 18.32 of 32), and participants completed on average 35.52 training sessions. Adverse events were reported infrequent (7.1%).

**Discussion and Conclusions:**

PROTECTapp is a promising mobile-based intervention to reduce PUI and enhance motivation to change in young adults. Its brevity, scalability, and safety profile highlight its potential as a low-threshold preventive or adjunctive intervention for young individuals at-risk.

## Introduction

The widespread integration of digital technologies into everyday life has been associated with an increase in impulse-driven and compulsive patterns of internet usage. The clinical relevance of the problematic usage of the internet (PUI) was recently acknowledged within the 11th revision of the International Classification of Diseases by including (online) gambling and gaming disorders within the category of disorders due to addictive behaviors (ICD-11; World Health Organization, 2018). Moreover, accumulating evidence suggests that other online behaviors—such as problematic use of pornography, buying/shopping, and social media use—may also constitute potential candidates for inclusion under the broader construct of internet use disorders (IUD; Brand et al., 2020). In contrast to specified IUDs, PUI more generally describes a pattern of excessive internet engagement independent of the respective medium but characterized by marked functional impairment (Fineberg et al., 2022). PUI has been linked to the deterioration of mental health among adolescents and young adults (McGorry et al., 2024). Among others, university students appear to be at particular risk given their elevated levels of smartphone use, with recent meta-analytic estimates indicating a prevalence of PUI of 11.3% (Amez, Vujić, De Marez, & Baert, 2023; Li et al., 2018). Within this population, PUI has been linked to adverse mental health outcomes and impaired academic functioning (Amez et al., 2023; Cai et al., 2023). While psychological interventions have demonstrated efficacy in the treatment of IUD (Goslar, Leibetseder, Muench, Hofmann, & Laireiter, 2020), further development of evidence-based, scalable, and cost-effective intervention approaches is needed to provide additional treatment options. Given their high acceptability among younger populations (Lincke et al., 2022), internet- and mobile-based interventions (IMIs) represent a particularly promising approach in this context; however, empirical evidence on the efficacy of IMIs for PUI remains limited (Boumparis et al., 2022).

Theoretical frameworks have highlighted the role of biased cognitive processing in the onset and maintenance of both PUI and IUD (Brand, Young, Laier, Wölfling, & Potenza, 2016, 2019; Liu et al., 2024). In particular, approach bias refers to an automatic action tendency to approach disorder-related cues, which – when stronger than or in conflict with concurrent avoidance tendencies (Breiner, Stritzke, & Lang, 1999) – has been linked to heightened addictive and other psychopathological symptomatology (Loijen, Vrijsen, Egger, Becker, & Rinck, 2020). The approach–avoidance task (AAT) is a well-established paradigm for assessing the approach bias. Within this paradigm, reaction times during approach (e.g., pulling a joystick) and avoidance (e.g., pushing a joystick) responses to both disorder-related and neutral stimuli (e.g., images) are measured. Faster reaction times during compatible trials (i.e., approach disorder-related stimuli and avoid neutral stimuli) compared to incompatible trials (i.e., avoid disorder-related stimuli and approach neutral stimuli) indicate approach bias (Rinck & Becker, 2007).

Cognitive biases are important targets for interventions designed to attenuate symptom severity by modifying maladaptive information-processing patterns. Empirical evidence indicates that a training variant of the AAT, which involves repeated exposure to incompatible trials, effectively reduces approach biases and associated symptoms of Internet Gaming Disorder (IGD) and other psychopathological conditions (He, Pan, Nie, Zheng, & Chen, 2021; Rabinovitz & Nagar, 2015; for overview, see Loijen et al., 2020). Based on the original joystick paradigm (Rinck & Becker, 2007), internet- and mobile-based adaptations of AAT trainings have been successfully implemented for targets such as cigarette smoking (Wittekind, Feist, Schneider, Moritz, & Fritzsche, 2015), body dissatisfaction (Kollei, Lukas, Loeber, & Berking, 2017), and procrastination (Lukas & Berking, 2018). However, findings on mobile-based approach bias modification for PUI remains scarce. In a randomized controlled pilot trial involving adolescents and young adults with elevated levels of PUI, a 3-week mobile-based AAT training was superior to a waitlist (WL) control group in reducing PUI, yielding a large between-group effect size (Lindenberg, Huth, Ebner, Schultze, & Brand, 2026).

However, these findings need to be interpreted as preliminary due to methodological limitations inherent to pilot studies (i.e., small sample size, restricted selection of secondary outcomes, lack of follow-up assessment). Therefore, the present prospective, two-armed, randomized controlled trial (RCT) aimed to corroborate preliminary evidence of the preceding pilot trial on the efficacy of a mobile-based AAT training for PUI (PROTECTapp) in young adults with elevated levels of pre-existing PUI. Our primary hypothesis was that PROTECTapp would be more efficacious in reducing PUI and internet-related craving, as assessed at baseline and post-intervention, compared to a waitlist (WL) control group. Furthermore, we explored effects of PROTECTapp on secondary internet-related outcomes, internalizing psychopathology, psychological well-being, and academic functioning (procrastination, self-efficacy). Finally, we aimed to explore the medium-term maintenance of treatment effects, adherence to the intervention, user satisfaction, and the occurrence of adverse effects.

## Methods

### Transparency and openness

Data and analysis code to reproduce main findings are available via PsychArchives at https://doi.org/10.23668/psycharchives.21767 (data) and https://doi.org/10.23668/psycharchives.21768 (code). Data were analyzed using R, version 4.5.1 (R Core Team, 2025). This study was pre-registered at the German Clinical Trials Register (DRKS00034109, May 8, 2024).

### Design

A 2-arm RCT was conducted to evaluate our hypotheses. Participants were randomly assigned to either (1) a 3-week mobile-based AAT training for PUI or (2) a 3-week WL control group. Online assessments were administered at baseline (T1) and immediately following the intervention or waitlist period (T2; post). Participants in the intervention group additionally completed follow-up assessments at 3 weeks (T3) and 12 weeks (T4) post-intervention. Following the waitlist period, participants in the WL control group received access to the AAT training and also completed post-intervention and follow-up assessments. However, these data are not reported within the present study, as they were deemed outside the scope of the present research objectives. Randomization was performed using permuted blocks with varying block sizes (2, 4, and 6) and a 1:1 allocation ratio. The randomization sequence was generated by the first author using R (version 4.3.3) and the randomizeR package (Uschner, Schindler, Hilgers, & Heussen, 2018). Neither participants nor study staff was blinded to the study conditions due to the nature of the inactive comparator. Allocation was concealed until completion of the baseline assessment. As subsequent assessments were conducted via self-administered online questionnaires, assessor blinding was not applicable at post-intervention and follow-up. Participants were compensated for provision of complete data either with course credit or by entering a lottery to win one of five €20 online shopping vouchers. There was no compliance-based compensation in association with app usage. We used the Consolidated Standards of Reporting Trials (CONSORT) reporting guideline.

### Participants

We recruited adult university students with elevated levels of PUI between May 10, 2024 and October 25, 2024. Participants were recruited through the university's website, mailing lists, and social media platforms. Inclusion criteria were: age ≥18 years, elevated levels of PUI as indicated by a score ≥24 in the Compulsive Internet Use Scale (CIUS; Meerkerk, Van Den Eijnden, Vermulst, & Garretsen, 2009), enrollment as a university student, ownership of a smartphone with internet access, sufficient German language knowledge, and provision of written informed consent. The chosen cut-off for PUI is commonly used to identify high-risk individuals, and has demonstrated a sensitivity of at least 70% for detecting cases of unspecified IUD (Bischof, Bischof, Meyer, John, & Rumpf, 2013). No further exclusion criteria were applied.

#### A priori power analysis

We performed an a priori power analysis based on an analysis of covariance (ANCOVA) comparing post-intervention scores between study groups and including baseline scores as covariate. To detect an expected medium-to-large between-group effect (*d* ≥ 0.65), based on previous evidence on mobile-based AAT trainings for PUI (Lindenberg et al., 2026) and other problem behaviors (Kollei et al., 2017; Lukas & Berking, 2018), a required sample size of *N* = 77 was determined, assuming a power of 80% and *α*-level of .05. To account for an anticipated dropout rate of 20%, the target sample size was increased to 92.

### Procedure

All study procedures were conducted online without any physical contact between the participants and the study staff. Data collection was conducted online using SoSci Survey (Leiner, 2024) and within the mobile application.

Interested individuals were screened for eligibility prior to study enrollment. Following the online screening procedure, eligible participants were provided with detailed written information regarding study procedures and gave written informed consent including contact information to schedule an introductory video conference for study enrollment. During this session, participants completed baseline assessments (T1) and were subsequently informed about group allocation (i.e., group allocation was carried out prior to the video conference but concealed until completion of the baseline assessment). Participants in the intervention group continued the introductory session and selected stimuli for the AAT training, and installed and pre-tested the mobile application. Participants allocated to the WL control group concluded the session after disclosure of group allocation and scheduled a second appointment to occur three weeks later. During this second session, WL participants completed post-intervention assessment (T2) and subsequently underwent the same preparatory procedures for the AAT training as the intervention group. In both groups, follow-up assessments administered outside of the video conferences were delivered automatically via email.

### Treatment conditions

#### PROTECTapp condition

The intervention was first described in more detail in Lindenberg et al. (2026). In brief, the PROTECTapp is a mobile-based ATT training designed to reduce PUI by modifying automatic action tendencies toward internet-related stimuli. During the study period, the PROTECTapp was freely accessible via the Apple App Store and Google Play Store, but activation of the application required a QR code that was provided exclusively to enrolled study participants. The PROTECTapp has not been commercially distributed, and the authors declare no financial or commercial interests related to the application. Participants were instructed to use the app for 21 consecutive days, completing at least three training sessions per day. To support adherence, participants were also required to set at least one daily reminder within the application. These reminders were individually scheduled by participants selecting the timing for notification delivery. The frequency of reminders was not adaptive to engagement with the intervention. Each training session consisted of 60 trials in which users swiped images representing functional (offline) activities downward to indicate approach and images representing dysfunctional (internet-related) activities upward to indicate avoidance, with immediate feedback on response accuracy provided after each trial. No additional reinforcement strategies (e.g., tokens, badges) were implemented. For one participant, training sessions comprised 100 trials due to unknown technical issues. To address the heterogeneity of PUI, participants selected 30 images (15 functional, 15 dysfunctional) from a pre-selected set of 340 images based on personal relevance (cf. Lindenberg et al., 2026). Dysfunctional stimuli within this standardized stimulus pool primarily reflected problematic gaming, problematic social networks use, and problematic mobile phone use. The stimulus pool did not include stimuli related to other online behaviors such as online gambling, online shopping, or pornography use. During the selection process, each image was presented for 2.5 s, and participants rated its personal relevance on an 11-point scale ranging from 0 (“the image is not relevant to me”) to 10 (“the image is highly relevant to me”). In each training session, the selected images were presented twice in randomized order.

#### Waitlist control condition

The WL control group underwent a three-week waiting period between baseline and post-intervention assessments. Following the post-intervention assessment, participants received access to the intervention and completed the same procedures and followed the same instructions as those in the intervention group.

### Measures

#### Primary outcomes

Primary outcomes of the present study were severity of PUI and internet-related craving. PUI was assessed using the Compulsive Internet Use Scale (CIUS; Meerkerk et al., 2009; German version: Wartberg, Petersen, Kammerl, Rosenkranz, & Thomasius, 2014). The CIUS comprises 14 items that evaluate core dimensions of PUI, including loss of control, withdrawal symptoms, coping, preoccupation, and interpersonal conflicts. Each item is rated on a 5-point Likert scale ranging from 0 (“never”) to 4 (“very often”). A total sum score is computed (possible range: 0–56), with higher scores indicating greater severity of PUI (Meerkerk et al., 2009; Peukert et al., 2012; Wartberg et al., 2014). The CIUS has demonstrated robust psychometric properties in prior studies (Gürtler et al., 2015; Peukert et al., 2012; Wartberg et al., 2014), and is recommended for screening of unspecified IUD by national diagnostic guidelines (Rehbein et al., 2025). In the present sample, the scale exhibited good internal consistency, as indicated by a McDonald's omega of *ω* = 0.87.

In line with theoretical models linking approach biases to craving (Brand et al., 2016), internet-related craving was additionally assessed using a self-developed modified version of the Gambling Craving Scale (GACS; Young & Wohl, 2009), hereafter referred to as the *Internet Craving Scale* (ICS). The ICS includes nine items assessing craving responses with respect to participants' most frequently used internet activity. Responses are provided on a 5-point Likert scale ranging from 0 (“strongly disagree”) to 4 (“strongly agree”). The original scale identified a three-factor structure—anticipation, desire, and relief—which demonstrated good internal consistencies (Young & Wohl, 2009). In the present study, confirmatory factor analysis supported this three-factor structure, indicated by good to acceptable fit indices according to Hu and Bentler (1999), *χ*^2^(22) = 53.81, *p* < .001, RMSEA = 0.072, 90% CI [0.000, 0.122], CFI = 0.992, TLI = 0.987, SRMR = 0.085. Subscale reliabilities were acceptable to good, with McDonald's omega values of *ω* = 0.76 (anticipation), *ω* = 0.78 (desire), and *ω* = 0.81 (relief).

#### Secondary outcomes

Secondary outcomes included motivation to change, symptoms of specified IUDs, as well as indicators of psychological and academic functioning. Motivation to change was assessed using an adapted version of the Stages of Change Readiness and Treatment Eagerness Scale (iSOCRATES; te Wildt, 2018), comprising three subscales: Recognition (i.e., acknowledgement of having a problem; sample *ω* = 0.93), Ambivalence (i.e., uncertainty or mixed feelings about the need to change; *ω* = 0.68), and Taking Steps (i.e., active efforts toward making positive change; *ω* = 0.88). Symptoms of IUDs were measured with the Internet Gaming Disorder Scale (IGDS; *ω* = 0.88; Lemmens, Valkenburg, & Gentile, 2015; German version: Wartberg, Kriston, & Thomasius, 2017) and the Social Media Disorder Scale (SMDS; *ω* = 0.79; Van Den Eijnden, Lemmens, & Valkenburg, 2016; German version: Wartberg, Kriston, & Thomasius, 2020). To enhance sensitivity to change, the standard reference period of 12 months in the IGDS and SMDS was modified to a 3-week interval. Internalizing psychopathology was evaluated using the ultra-brief screener of the Patient Health Questionnaire (PHQ-4; *ω* = 0.85; Kroenke, Spitzer, Williams, & Löwe, 2009; German version: Löwe et al., 2010). Symptoms of social anxiety were assessed with the short version of the Social Phobia Inventory (mini-SPIN; *ω* = 0.82; Connor, Kobak, Churchill, Katzelnick, & Davidson, 2001; German version: Wiltink et al., 2017). Psychological well-being was measured via the 5-item World Health Organization Well-Being Index (WHO-5; *ω* = 0.83; World Health Organization, 1998). Procrastination was assessed using the short version of the Procrastination Questionnaire for Students (PFS-4; *ω* = 0.93; Glöckner-Rist, Engberding, Höcker, & Rist, 2009). General and study-related self-efficacy were evaluated using the General Self-Efficacy Scale (SWE; *ω* = 0.88; Schwarzer & Jerusalem, 2003) and its study-specific adaptation (WIRKSTUD; *ω* = 0.91), respectively.

#### Intervention usage and additional measures

App usage metrics were recorded within the mobile application, capturing count of training sessions, as well as reaction times and accuracy per trial within each training session. Sociodemographic data, including age, gender, relationship status, academic year, most frequently used online activity, and gaming as well as social media usage times were collected by self-report measures. Negative effects were assessed at post-intervention using an open response format.

### Statistical analysis

**Fig. 1.**
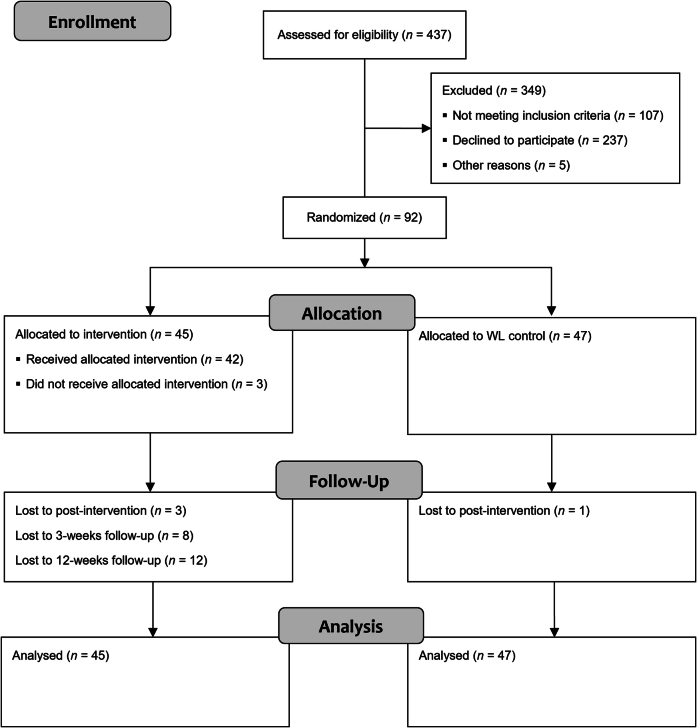
Participant flow *Note*. WL, waitlist control group

The hypothesized efficacy of the intervention was evaluated based on: (1) changes in participants' levels of PUI and internet-related craving, and secondary outcomes from baseline to post-intervention, (2) the number of participants demonstrating a treatment response, and (3) the number of participants exhibiting symptom deterioration. Analyses of primary and secondary outcomes were conducted according to the intention-to-treat (ITT) principle, incorporating all participants that were randomized to either study condition (see Fig. 1). Changes in primary and secondary outcomes between study conditions were examined using mixed models for repeated measures (MMRM) including group, time and the group × time interaction as fixed effects. Models were estimated using restricted maximum likelihood (REML), and longitudinal dependencies were accounted for using unstructured variance-covariance matrices. To address small-sample inference, the method by Kenward and Roger (1997) was applied. In instances where model assumptions (e.g., normality of residuals, homoscedasticity) were violated, sensitivity analyses were conducted based on the procedures outlined by Maruo, Ishii, Yamaguchi, Ohigashi, and Gosho (2024), and any deviations from the primary analyses are reported. Within the intervention group, the change in outcomes from baseline to the 12-week follow-up was examined with equivalent MMRMs including time (baseline, post-intervention, 3-week follow-up, 12-week follow-up) as a fixed effect. Between-group effect sizes (Cohen's *d*) were calculated using estimated mean differences at post-intervention and pooled observed standard deviations (*SD*s) at post-intervention. Within-group effect sizes were calculated using estimated mean pre-post differences divided by the observed *SD* at the respective follow-up. According to Cohen (1988), *d* = 0.2 represents a small effect, *d* = 0.5 a medium effect, and *d* = 0.8 a large effect.

To determine the number of participants achieving reliable improvement in PUI, participants were classified as responders or non-responders based on the Reliable Change Index (RCI; Jacobson & Truax, 1992). Using the test-retest reliability of the CIUS (Kuzucu, Ozdemir, & Ak, 2015) and the baseline sample *SD*, a reduction of more than 6.08 points on the CIUS from baseline to post-intervention was considered indicative of treatment response. Negative effects of the intervention were assessed by identifying participants with reliable symptom deterioration in PUI according to the RCI. Chi-square tests were performed to compare frequencies of reliable change between study groups.

We performed sensitivity analyses in a subgroup of participants who were adherent to the intervention per protocol (i.e., ≥21 training sessions, corresponding to one third of the recommended training dose) and provided complete data at post-intervention. Descriptive statistics of the total number of completed training sessions were calculated to explore app usage among the intervention group. We examined associations of number of training sessions with primary outcomes at post-intervention controlling for respective baseline values. Changes in AAT performance were assessed using paired samples *t*-tests comparing mean reaction times between the first and last session, as well as linear mixed models (LMMs) predicting log-transformed mean reaction times from session, condition (i.e., approach vs avoidance gesture), and their interaction. Random effects were specified for session and participant. Before computing mean values, we excluded the first training session, all erroneous trials, trials with reaction times <150 ms or >2000 ms, and with reaction times deviating by ≥ 2 SDs from session- and condition-specific means (cf. Kahveci, Rinck, Van Alebeek, & Blechert, 2023).

### Ethics

The study procedures were carried out in accordance with the Declaration of Helsinki. The local ethics committee of the Department of Psychology, University of Mainz, Germany (reference number 2024-JGU-psychEK-016, May 6, 2024) approved the study. All subjects were informed about the study and all provided informed consent.

## Results

### Sample characteristics

During the recruitment phase, a total of 437 individuals were screened for eligibility, of whom 92 were consecutively enrolled and randomized to one of the study conditions (PROTECTapp: *n* = 45; WL: *n* = 47; see Fig. 1). The rate of attrition at post-intervention was 4.3% (*n* = 4). Within the intervention group, 6.7% (*n* = 3) of participants did not initiate the AAT training, including one individual who was unable to access the intervention.

Sociodemographic characteristics of the sample are presented in Table 1. Participants had a mean age of 22.00 years (*SD* = 2.66), with 69.6% (*n* = 64) identifying as women. On average, participants had completed 5.10 semesters (*SD* = 3.32). With respect to participant's most frequently used online activity, the majority reported social media use (*n* = 49, 53.3%), followed by streaming of series, movies, or music (*n* = 21, 22.8%), YouTube or Twitch use (*n* = 19, 20.7%), and video gaming (*n* = 3, 3.3%). Daily engagement with digital media comprised on average 0.88 h (*SD* = 1.39) spent gaming and 2.89 h (*SD* = 1.97) spent on social media. Primary and secondary outcomes at baseline are reported in Supplementary Table S1. The average level of PUI (CIUS) was elevated, however, mean scores on internalizing psychopathology (PHQ-4) remained below clinically significant thresholds. No significant between-group differences were observed at baseline for sociodemographic variables or primary and secondary outcome measures (*ps* > .05).

**Table 1.** Sample characteristics at baseline

Characteristic	Total sample (92)	PROTECTapp (45)	WL (47)	Test statistic
Age, years, *M*(*SD*)	22.00 (2.66)	21.93 (2.90)	22.06 (2.44)	*t*(90) = 0.23, *p* = .816
Gender^1^, *n*(%)				*χ*^2^ = 2.13, *p* = .314
Women	64 (69.6)	34 (75.6)	30 (63.8)	
Men	27 (29.3)	11 (24.4)	16 (34.0)	
Other	1 (1.1)	–	1 (2.1)	
Relationship status^1^, *n*(%)				*χ*^2^ = 1.52, *p* = .527
Single	55 (59.8)	25 (55.6)	30 (63.8)	
In relationship	36 (39.1)	19 (42.2)	17 (36.2)	
Married	1 (1.1)	1 (2.2)	–	
Living situation^1^, alone, *n*(%)				*χ*^2^ = 1.43, *p* = .714
Alone	28 (30.4)	16 (35.6)	12 (25.5)	
With non-relatives	42 (45.7)	20 (44.4)	22 (46.8)	
With partner	9 (9.8)	4 (8.9)	5 (10.6)	
With parents	13 (14.1)	5 (11.1)	8 (17.0)	
Academic semester, *M*(SD)	5.10 (3.32)	4.56 (3.11)	5.62 (3.46)	*t*(90) = 1.55, *p* = .126
Daily time spent gaming, hours, *M*(*SD*)	0.88 (1.39)	0.71 (0.99)	1.05 (1.68)	*t*(90) = 1.19, *p* = .238
Daily time spent on social media, hours, *M*(*SD*)	2.89 (1.97)	2.83 (1.79)	2.95 (2.16)	*t*(90) = 0.27, *p* = .786

*Note*. PROTECTapp, mobile-based approach-avoidance task training for problematic internet use, WL, waitlist control group. ^1^Chi-squared test with simulated *p*-value (based on 5,000 replicates).

### Primary outcomes

**Fig. 2.**
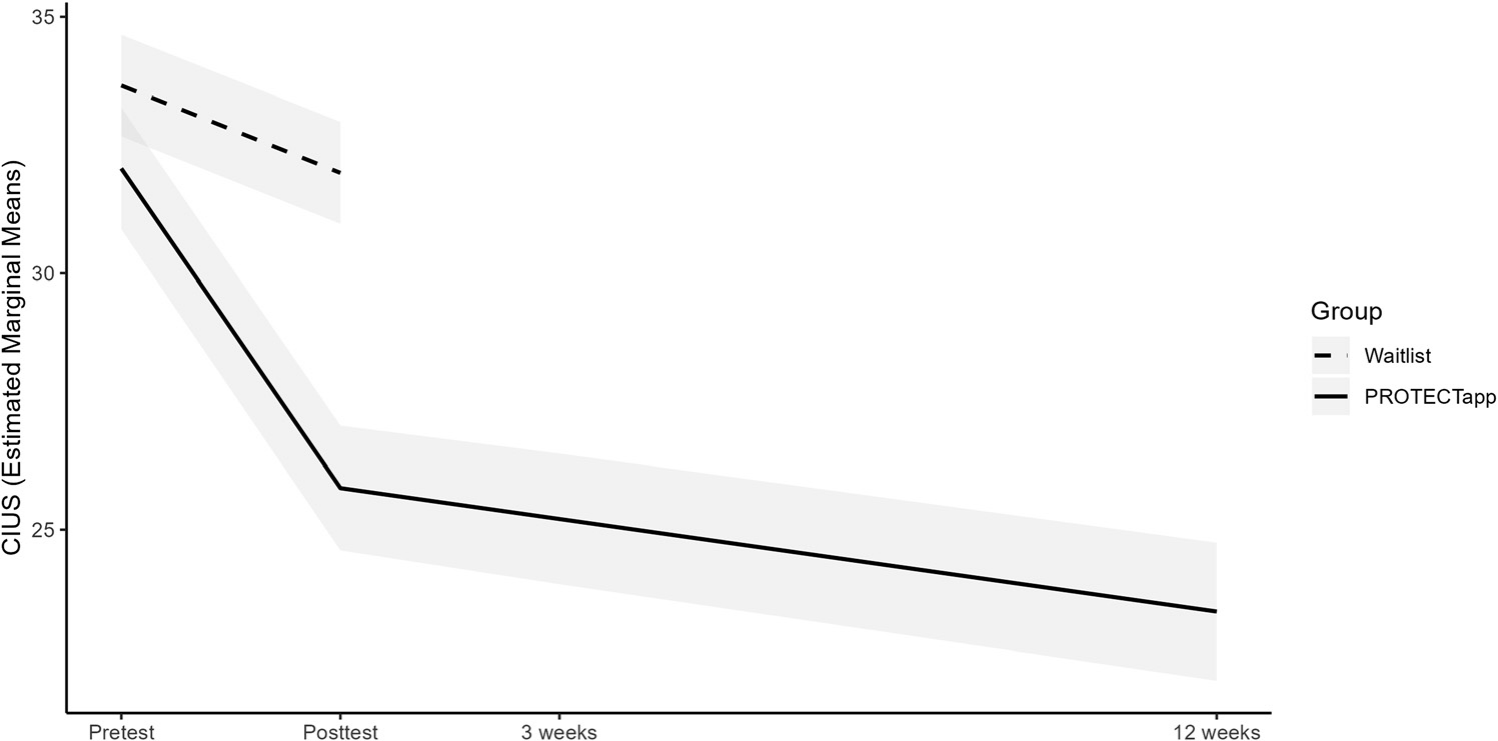
Changes of mean scores of problematic use of the internet (CIUS) across time *Note*. CIUS, Compulsive Internet Use Scale, PROTECTapp, mobile-based approach-avoidance task training for problematic internet use

Consistent with our hypothesis, ITT analyses revealed a significant time × group interaction for the CIUS, *b* = −4.54, SE = 1.44, *p* = .003, indicating a greater reduction of PUI in the intervention group compared to the WL control group over 3 weeks (see Fig. 2), with a medium-to-large between-group effect at post-intervention, *d* = −0.80, 95% CI [−1.23, −0.38]. Among participants with complete data (*n* = 88), a significantly higher proportion of participants in the intervention group achieved a reliable improvement on the CIUS compared to the WL control group (19/42 [45.2%] vs 4/46 [8.7%]), *χ*^2^ = 15.19, *p* < .001 over 3 weeks. In contrast, no significant time × group interactions were observed for any of the ICS subscales (*p*s > .05; see Table 2).

**Table 2.** Estimated means and observed SDs at baseline and post-intervention, model-based group × time interaction effects, and effect sizes for primary outcomes for the intention-to-treat sample

Measure (possible range)	Baseline	Post-intervention	Effect size *d* [95% CI]	
	*M*(*SD*)	*M*(*SD*)	Within (Pre/Post)	Between (Post)
Problematic internet use, CIUS (0–56)	*Interaction effect*: *b* = −4.54, *SE* = 1.44, *p* = .003			
PROTECTapp	32.04 (6.72)	25.80 (8.14)	−0.77 [−1.10, −0.43]	−0.80 [−1.23, −0.38]
WL	33.66 (6.49)	31.96 (7.24)	−0.23 [−0.52, 0.06]	
Internet craving, anticipation, ICS (0–12)	*Interaction effect*: *b* = −1.05, *SE* = 0.60, *p* = .084			
PROTECTapp	7.98 (2.21)	6.67 (2.64)	−0.50 [−0.81, −0.19]	−0.41 [−0.82, 0.00]
WL	8.09 (2.54)	7.82 (2.96)	−0.09 [−0.38, 0.20]	
Internet craving, desire, ICS (0–12)	*Interaction effect*: *b* = 0.11, *SE* = 0.52, *p* = .836			
PROTECTapp	3.22 (2.40)	3.30 (2.24)	0.03 [−0.26, 0.33]	−0.14 [−0.55, 0.27]
WL	3.66 (2.32)	3.63 (2.55)	−0.01 [−0.30, 0.27]	
Internet craving, relief, ICS (0–12)	*Interaction effect*: *b* = 0.50, *SE* = 0.49, *p* = .317			
PROTECTapp	2.53 (2.61)	2.51 (2.28)	−0.01 [−0.30, 0.28]	−0.01 [−0.41, 0.40]
WL	3.04 (2.62)	2.52 (2.47)	−0.21 [−0.50, 0.08]	

*Note*. PROTECTapp, mobile-based approach-avoidance task training for problematic internet use, WL, waitlist control group, CIUS, Compulsive Internet Use Scale, ICS, Internet Craving Scale.

### Secondary outcomes

ITT analyses of secondary outcomes revealed significant time × group interaction effects in favor of the intervention on the Ambivalence (*b* = −1.08, *SE* = 0.45, *p* = .020) and Taking Steps (*b* = 3.58, *SE* = 1.09, *p* = .002) subscales of the iSOCRATES. At post-intervention, between-group effect sizes were small-to-medium, with *d* = −0.24, 95% CI [−0.65, 0.17] for Ambivalence and *d* = 0.45, 95% CI [0.04, 0.87] for Taking Steps. No significant time × group interactions were observed for any other secondary outcomes (*ps* > .05; see Supplementary Table S2).

### Subgroup analyses

Per-protocol (PP) analyses of 28 participants (62.2%) with complete baseline and post-intervention data who completed ≥21 AAT training sessions showed significantly greater improvements, compared to the WL control group (*n* = 46), on the CIUS, *b* = −4.27, SE = 1.70, *p* = .017, between-group effect size: *d* = −0.92, 95% CI [−1.41, −0.43], and Taking steps subscale of the iSOCRATES, *b* = 3.22, SE = 1.16, *p* = .008, between-group effect size: *d* = 0.43, 95% CI [−0.05, 0.90]. No significant time × group interactions were found for the remaining outcome variables within this subsample (*p*s > .05). PP participants significantly differed from drop-outs regarding age (PP: *M*(*SD*) = 21.62 (2.48), drop-out: *M*(*SD*) = 23.67 (2.83), *p* = .003), semesters studied (PP: *M*(*SD*) = 4.74 (3.29), drop-out: *M*(*SD*) = 6.56 (3.13), *p* = .037), and procrastination (PP: *M*(*SD*) = 12.34 (4.45), drop-out: *M*(*SD*) = 14.89 (3.61), *p* = .027). Detailed results of PP analyses can be found in Supplementary Table S3.

### Follow-up assessment

Participants in the intervention group demonstrated significant improvements from baseline to the 12-week follow-up on multiple internet-related outcomes (Fig. 2). Specifically, improvements were observed on the CIUS (*p* < .001, *d* = −0.98 [−1.40, −0.57]), the Anticipation subscale of the ICS (*p* = .013, *d* = −0.42 [−0.77, −0.06]), the Ambivalence (*p* < .001, *d* = −0.86 [−1.26, −0.46]), and Taking Steps subscales of the iSOCRATES (*p* = .021, *d* = 0.38 [0.03, 0.74]), the IGDS (*p* < .001, *d* = −1.38 [−1.86, −0.90]), and the SMDS (*p* < .001, *d* = −0.65 [−1.02, −0.27]). However, a significant decrease was also noted on the Recognition subscale of the iSOCRATES (*p* < .001, *d* = −0.70 [−1.08, −0.32]). No other within-person changes from baseline to 12-week follow-up reached statistical significance (*ps* > .05). Detailed results can be found in Supplementary Table S4.

### Intervention usage and satisfaction

Among participants who initiated the AAT training, participants completed on average 35.52 training sessions (*SD* = 20.38, range: 3–68). Number of training sessions was not significantly associated with primary outcomes at post-intervention (*p*s > .05). Satisfaction with the PROTECTapp was moderate, with a total score of 18.32 (*SD* = 5.23) out of 32.

Changes in performance were analyzed within the intervention group with pre-post comparisons and a LMM predicting log-transformed reaction times from session, condition (approach vs avoidance), and their interaction. When comparing mean reaction times between first and last training session, we observed reduced reaction times in both approach, *M*_pre_ = 880.45 ms, *SD*_pre_ = 117.29, *M*_post_ = 792.95 ms, *SD*_post_ = 114.47, *p* < .001, *d* = −0.76 [−0.95, −0.28], and avoidance conditions, *M*_pre_ = 877.50 ms, *SD*_pre_ = 129.53, *M*_post_ = 788.29 ms, *SD*_post_ = 119.41, *p* < .001, *d* = −0.75 [−1.05, −0.37]. Similarly, LMM results revealed a significant linear effect of session, *b* < 0.01, SE < 0.01, *p* = .002, but not for condition or their interaction (*p*s > .05).

### Negative effects

Three participants (7.1%) reported negative effects of the AAT training including continued smartphone usage after training sessions (*n* = 2), and the experience of the AAT training as monotonous and boring (*n* = 1). Additionally, reliable deterioration of PUI (CIUS) was observed in 4 participants in the intervention group (9.5%), and 2 participants in the WL control group (4.3%). Frequencies of reliable deterioration did not differ significantly between study conditions, *χ*^2^ = 0.93, *p* = .424.

## Discussion and conclusions

The present RCT evaluated the efficacy of a mobile-based AAT training (PROTECTapp) for reducing PUI in young adults. Consistent with our hypothesis, participants in the intervention group exhibited significantly greater reductions in PUI relative to the WL control group following three weeks, corresponding to a medium-to-large between-group effect size. Nearly half of the intervention group achieved reliable improvement, compared to less than 10% in the WL control group. Between-group effect sizes were more pronounced among participants adherent to the study protocol. Beyond PUI, the intervention yielded favorable effects on motivation to change. Within-group analyses suggested maintenance of PUI reduction and improvements in certain IUD-specific symptoms up to 12 weeks post-intervention. These findings extend preliminary pilot data and add robust evidence that mobile-based AAT trainings may be an effective and scalable strategy for addressing PUI in younger cohorts (Lindenberg et al., 2026). The observed effects align with theoretical models positing that biased approach tendencies toward internet-related cues contribute to the onset and maintenance of internet-related addictive behaviors (Brand et al., 2016; Breiner et al., 1999). By systematically pairing internet-related stimuli with avoidance movements, the intervention may have attenuated maladaptive stimulus–response associations, thereby facilitating behavioral change. Similar mechanisms have been demonstrated across addictive and other mental health conditions (Loijen et al., 2020), and successful modification has previously been achieved using internet- and mobile-based delivery formats (Kollei et al., 2017; Lukas & Berking, 2018; Wittekind et al., 2015). The observed effect of the PROTECTapp on PUI was descriptively larger compared to other mobile-based interventions targeting mental health problems, which rely mostly on different mechanisms of change (Linardon, Cuijpers, Carlbring, Messer, & Fuller-Tyszkiewicz, 2019; Weisel et al., 2019).

However, no significant between-group differences emerged for internet-related craving. This finding contradicts previous evidence on the reduction of craving on other appetitive cues through AAT trainings (Kakoschke, Kemps, & Tiggemann, 2017). A possible explanation may be that, although the intervention was effective in modifying problematic usage patterns, it might simultaneously have maintained craving, as it also represents a cue associated with the addictive behavior. Another explanation could be that the measurement of internet-related craving was inadequate. We relied on an adapted version of a measure originally designed for gambling-related craving (Young & Wohl, 2009), which has not yet been validated for PUI. Although preliminary analyses support the factorial validity, comprehensive psychometric validation of the adapted instrument is still pending. Moreover, the heterogeneity of unspecified PUI may obscure effects on craving when assessed at a general level. Parallelizing research on specified IUDs may thus provide a more sensitive index for capturing craving-related changes (e.g., Rabinovitz & Nagar, 2015).

Consistent with previous studies (He et al., 2021; Lindenberg et al., 2026), secondary outcomes unrelated to internet use (e.g., psychological well-being, academic functioning) did not differ significantly between conditions. The absence of significant improvements in psychological well-being may be attributable to several factors. First, although approach bias modification has shown promise for other psychological symptoms (Loijen et al., 2020), the stimulus material likely needs to be domain-specific to the targeted symptomatology. Second, the relatively low level of psychopathological symptom distress in the present sample may have introduced floor effects. Third, broader cognitive-emotional processes were not directly targeted by the PROTECTapp. We propose that reductions in PUI may constitute a prerequisite for subsequent improvements in psychological well-being, but modification of related affective processes may require longer training phases or integration within blended intervention strategies.

Findings of the present study corroborate the feasibility of the PROTECTapp. The intervention demonstrated a favorable safety profile characterized by low reported rates of adverse events and no evidence of systematic symptom deterioration. Mean satisfaction with and adherence to the training was moderate. In line with previous findings on low long-term engagement with mental health mobile applications (Baumel, Muench, Edan, & Kane, 2019), adherence substantially varied across participants, while total number of training sessions was not significantly associated with pre-post changes in PUI. Evidence regarding changes in training performance, however, indicated slight increases in speed for both approach and avoidance trials. The absence of a significant dose-response relationship aligns with prior evidence suggesting that approach bias modification in IGD can be achieved after only a few training sessions (He et al., 2021; Rabinovitz & Nagar, 2015). However, the pattern of results warrants caution when concluding about approach bias modification as the primary mechanism of change. Although previous research supports the assumption that the modification of biased approach tendencies underlies symptom improvement (Kakoschke et al., 2017), the design of the present study does not permit robust conclusions as direct measurement of the approach bias was not implemented and changes in reaction times on incompatible trials can at best be considered a proxy. Concerns are further supported by findings that sham AAT trainings have been equally effective in reducing symptoms of alcohol consumption and gambling (Wiers et al., 2015; Wittekind et al., 2019). Future studies should incorporate approach bias assessment as an outcome and employ modified AAT variants as active control conditions to clarify the mechanisms underlying symptom change.

Further limitations should be noted. Because the study did not recruit a clinically diagnosed sample, the generalizability of the findings to clinical populations with IUD is limited. Instead, the PROTECTapp was evaluated as a low-threshold preventive intervention for an at-risk population rather than as a stand-alone treatment strategy. Generalizability is further constrained by the recruitment of university students, which limits conclusions regarding younger individuals and those with lower educational attainment. Although the sample was approximately representative of German university students with respect to mean age (Kroher et al., 2021), the disproportionately higher inclusion of women likely biased the distribution of PUI toward a greater prevalence of problematic social media use relative to other types (e.g., internet gaming; Fineberg et al., 2022). This assumption is supported by the high proportion of participants reporting social media and streaming services as their most frequently used online activities, as well as by a post-hoc evaluation of the 15 most frequently selected dysfunctional stimuli, which exclusively reflected problematic social media and mobile phone use. Moreover, the absence of stimuli depicting online gambling, online shopping, or pornography use further limits the generalizability of the findings across the full range of online behaviors subsumed under the construct of PUI. The absence of blinding may have introduced expectancy effects. Given that participants' contingency awareness possibly moderates the efficacy of AAT trainings (Van Dessel, De Houwer, & Gast, 2015), disclosure of the interventions' goals and procedures may have amplified training effects. Furthermore, the use of a WL control group as a comparator combined with procedural differences between groups (i.e., conclusion of the introductory session after disclosure of group allocation in the WL control group) may have amplified disappointment effects, thereby increasing the risk of overestimating the intervention's efficacy. Additional methodological limitations include the reliance on self-report measures, and the inability to compare long-term effects between groups. Finally, the relatively extensive process of stimuli selection may itself have exerted an influence, potentially confounding training effects attributable specifically to the AAT training.

Overall, the present RCT provides further support that a brief, mobile-based approach bias modification training can produce meaningful reductions in PUI and enhance readiness to change among young adults. While effects on craving and other secondary outcomes were less consistent, benefits persisted up to 12 weeks post-intervention. Given its brevity, scalability, and minimal resource requirements, the PROTECTapp holds promise as a low-threshold preventive or adjunctive intervention for young individuals at risk for PUI.

## Supplementary material
