# Supplementary material for: Efficacy of a mobile-based approach-avoidance task training (PROTECTapp) for problematic usage of the internet in young adults: A randomized controlled trial
Source: J Behav Addict. 2026 Apr 7;15(2):812–23. doi: 10.1556/2006.2025.00551 (PMC13371780; doi:10.1556/2006.2025.00551)
Supplement: Supplementary file 1 [file jba-15-812-s001.pdf]

Huth, D., Brand, S., & Lindenberg, K.: Efficacy of a mobile-based approach-avoidance task training (PROTECTapp) for problematic usage of the internet in young adults: A randomized controlled trial

https://doi.org/10.1556/2006.2025.00551

Supplementary materials

CONTENT

Table S1. *Observed means and SDs of primary and secondary outcomes at baseline*

Table S2. *Estimated means and observed SDs at baseline and post-intervention, model-based group × time interaction effects, and effect sizes for secondary outcomes for the intention-to-treat sample*

Table S3. *Estimated means and observed SDs at baseline and post-intervention, model-based group × time interaction effects, and effect sizes for primary and secondary outcomes for the per-protocol subsample*

Table S4. *Estimated means and observed SDs at follow-up assessments, model-based effects of time, and within-person effect sizes for primary and secondary outcomes in the intervention group (intention-to-treat sample)*

Table S1

Observed means and SDs of primary and secondary outcomes at baseline

Measure (possible range)	Total sample (92)	PROTECTapp (45)	WL (47)	Test statistic
Problematic internet use, CIUS (0-56), *M*(*SD*)	32.87 (6.62)	32.04 (6.72)	33.66 (6.49)	*t*(90) = 1.17, *p* = .244
Internet-related craving, anticipation, ICS (0-12), *M*(*SD*)	8.03 (2.37)	7.98 (2.21)	8.09 (2.54)	*t*(90) = 0.22, p = .829
Internet-related craving, desire, ICS (0-12), *M*(*SD*)	3.45 (2.36)	3.22 (2.40)	3.66 (2.32)	*t*(90) = 0.89, p = .376
Internet-related craving, relief, ICS (0-12), *M*(*SD*)	2.79 (2.61)	2.53 (2.61)	3.04 (2.62)	*t*(90) = 0.93, p = .353
Motivation to change, recognition, iSOCRATES (7-35), *M*(*SD*)	22.87 (5.75)	23.00 (5.73)	22.74 (5.82)	*t*(90) = -0.21, p = .833
Motivation to change, ambivalence, iSOCRATES (4-20), *M*(*SD*)	13.86 (2.39)	14.04 (2.18)	13.68 (2.58)	*t*(90) = -0.73, *p* = .469
Motivation to change, taking steps, iSOCRATES (8-40), *M*(*SD*)	23.55 (5.64)	23.18 (5.74)	23.91 (5.58)	*t*(90) = 0.62, *p* = .534
Internet gaming disorder symptoms, IGDS (0-9), *M*(*SD*)	1.40 (1.93)	1.16 (1.64)	1.64 (2.16)	*t*(90) = 1.20, *p* = .232
Social media disorder symptoms, SMDS (0-9), *M*(*SD*)	2.26 (1.76)	2.11 (1.91)	2.40 (1.61)	*t*(90) = 0.80, *p* = .427
Internalizing symptoms, PHQ-4 (0-12), *M*(*SD*)	4.41 (2.78)	4.22 (2.50)	4.60 (3.03)	*t*(90) = 0.64, *p* = .522
Social anxiety symptoms, mini-SPIN (0-12), *M*(*SD*)	3.80 (2.86)	3.91 (2.70)	3.70 (3.04)	*t*(90) = -0.35, *p* = .729
Well-being, WHO-5 (0-25), *M*(*SD*)	11.91 (4.33)	12.42 (3.88)	11.43 (4.71)	*t*(90) = -1.11, *p* = .272
Procrastination, PFS-4 (4-20), *M*(*SD*)	12.84 (4.40)	12.91 (4.40)	12.77 (4.44)	*t*(90) = -0.16, *p* = .875
General self-efficacy, SWE (10-40), *M*(*SD*)	28.04 (4.60)	28.11 (4.15)	27.98 (5.04)	*t*(90) = -0.14, *p* = .891
Study-related self-efficacy, WIRKSTUD (7-28), *M*(*SD*)	18.89 (4.18)	18.98 (3.51)	18.81 (4.78)	*t*(84.37) = -0.19, *p* = .847

Note. PROTECTapp, mobile-based approach-avoidance task training for problematic internet use, WL, waitlist control group. CIUS, Compulsive Internet Use Scale, ICS, Internet Craving Scale, iSOCRATES, Stages of Change Readiness and Treatment Eagerness Scale (adapted for internet use), IGDS, Internet Gaming Disorder Scale, SMDS, Social Media Disorder Scale, PHQ-4, Patient Health Questionnaire – ultra-brief screener, mini-SPIN, Mini Social Phobia Inventory, WHO-5, WHO-5 Well-being Index, PFS-4, Procrastination Questionnaire for Students, SWE, General Self-Efficacy Scale, WIRKSTUD, Study-specific Self-Efficacy Scale.

Table S2

Estimated means and observed SDs at baseline and post-intervention, model-based group × time interaction effects, and effect sizes for secondary outcomes for the intention-to-treat sample

Measure (possible range)	Baseline	Post-intervention	Effect size *d* [95% CI]	
	M(*SD*)	*M*(*SD*)	Within (Pre/Post)	Between (Post)
Motivation to change, recognition, iSOCRATES (7-35)	*Interaction effect*: *b* = -0.98, *SE* = 0.75, *p* = .198			
PROTECTapp	23.00 (5.73)	21.52 (5.78)	-0.26 [-0.55, 0.04]	-0.12 [-0.52, 0.29]
WL	22.74 (5.82)	22.25 (6.68)	-0.07 [-0.36, 0.21]	
Motivation to change, ambivalence, iSOCRATES (4-20)	*Interaction effect*: *b* = -1.08, *SE* = 0.45, *p* = .020			
PROTECTapp	14.04 (2.18)	12.90 (2.86)	-0.40 [-0.70, -0.10]	-0.24 [-0.65, 0.17]
WL	13.68 (2.58)	13.61 (2.99)	-0.02 [-0.31, 0.26]	
Motivation to change, taking steps, iSOCRATES (8-40)	*Interaction effect*: *b* = 3.58, *SE* = 1.09, *p* = .002			
PROTECTapp	23.18 (5.74)	26.72 (6.36)	0.56 [0.24, 0.87]	0.45 [0.04, 0.87]
WL	23.91 (5.58)	23.88 (6.17)	-0.01 [-0.29, 0.28]	
Internet gaming disorder symptoms, IGDS (0-9)	*Interaction effect*: *b* = -0.29, *SE* = 0.34, *p* = .398			
PROTECTapp	1.16 (1.64)	0.71 (1.28)	-0.35 [-0.65, -0.05]	-0.48 [-0.90, -0.07]
WL	1.64 (2.16)	1.47 (1.85)	-0.09 [-0.38, 0.20]	
Social media disorder symptoms, SMDS (0-9)	*Interaction effect*: *b* = -0.44, *SE* = 0.34, *p* = .198			
PROTECTapp	2.11 (1.91)	1.38 (1.59)	-0.46 [-0.77, -0.15]	-0.46 [-0.88, -0.05]
WL	2.40 (1.61)	2.11 (1.59)	-0.18 [-0.47, 0.10]	

(to be continued)

Table S2 (*continued*)

Measure (possible range)	Baseline	Post-intervention	Effect size *d* [95% CI]	
	M(*SD*)	*M*(*SD*)	Within (Pre/Post)	Between (Post)
Internalizing symptoms, PHQ-4 (0-12)	*Interaction effect*: *b* = 0.36, *SE* = 0.44, *p* = .422			
PROTECTapp	4.22 (2.50)	4.86 (3.22)	0.20 [-0.10, 0.49]	0.00 [-0.41, 0.40]
WL	4.60 (3.03)	4.88 (3.10)	0.09 [-0.20, 0.38]	
Social anxiety symptoms, mini-SPIN (0-12)	*Interaction effect*: *b* = -0.44, *SE* = 0.40, *p* = .270			
PROTECTapp	3.91 (2.70)	4.39 (2.98)	0.16 [-0.13, 0.46]	-0.08 [-0.49, 0.33]
WL	3.70 (3.04)	4.63 (3.06)	0.30 [0.01, 0.59]	
Well-being, WHO-5 (0-25)	*Interaction effect*: *b* = -0.43, *SE* = 0.81, *p* = .594			
PROTECTapp	12.42 (3.88)	11.87 (5.17)	-0.11 [-0.40, 0.19]	0.11 [-0.30, 0.52]
WL	11.43 (4.71)	11.30 (4.89)	-0.03 [-0.31, 0.26]	
Procrastination, PFS-4 (4-20)	*Interaction effect*: *b* = -0.42, *SE* = 0.48, *p* = .387			
PROTECTapp	12.91 (4.40)	12.84 (3.74)	-0.02 [-0.31, 0.27]	-0.06 [-0.47, 0.35]
WL	12.77 (4.44)	13.11 (4.72)	0.07 [-0.21, 0.36]	
General self-efficacy, SWE (10-40)	*Interaction effect*: *b* = -1.22, *SE* = 0.62, *p* = .052			
PROTECTapp	28.11 (4.15)	27.29 (3.99)	-0.21 [-0.50, 0.09]	-0.23 [-0.64, 0.18]
WL	27.98 (5.04)	28.38 (5.24)	0.08 [-0.21, 0.36]	
Study-related self-efficacy, WIRKSTUD (7-28)	*Interaction effect*: *b* = 0.02, *SE* = 0.55, *p* = .969			
PROTECTapp	18.98 (3.51)	18.43 (3.61)	-0.15 [-0.45, 0.14]	0.05 [-0.36, 0.46]
WL	18.81 (4.78)	18.24 (4.25)	-0.13 [-0.42, 0.15]	

Note. PROTECTapp, mobile-based approach-avoidance task training for problematic internet use, WL, waitlist control group. CIUS, Compulsive Internet Use Scale, ICS, Internet Craving Scale, iSOCRATES, Stages of Change Readiness and Treatment Eagerness Scale (adapted for internet use), IGDS, Internet Gaming Disorder Scale, SMDS, Social Media Disorder Scale, PHQ-4, Patient Health Questionnaire – ultra-brief screener, mini-SPIN, Mini Social Phobia Inventory, WHO-5, WHO-5 Well-being Index, PFS-4, Procrastination Questionnaire for Students, SWE, General Self-Efficacy Scale, WIRKSTUD, Study-specific Self-Efficacy Scale.

Table S3

Estimated means and observed SDs at baseline and post-intervention, model-based group × time interaction effects, and effect sizes for primary and secondary outcomes for the per-protocol subsample

Measure (possible range)	Baseline	Post-intervention	Effect size *d* [95% CI]	
	M(*SD*)	*M*(*SD*)	Within (Pre/Post)	Between (Post)
Problematic internet use, CIUS (0-56)	*Interaction effect*: *b* = -4.27, *SE* = 1.70, *p* = .017			
PROTECTapp	31.07 (6.02)	25.11 (7.69)	-0.78 [-1.20, -0.35]	-0.92 [-1.41, -0.43]
WL	33.63 (6.56)	31.93 (7.24)	-0.23 [-0.53, 0.06]	
Internet-related craving, anticipation, ICS (0-12)	*Interaction effect*: *b* = -0.63, *SE* = 0.66, *p* = .344			
PROTECTapp	7.64 (2.34)	6.75 (2.30)	-0.39 [-0.77, 0.00]	-0.39 [-0.86, 0.09]
WL	8.07 (2.56)	7.80 (2.96)	-0.09 [-0.38, 0.20]	
Internet-related craving, desire, ICS (0-12)	*Interaction effect*: *b* = 0.27, *SE* = 0.62, *p* = .661			
PROTECTapp	2.89 (1.89)	3.14 (2.40)	0.10 [-0.27, 0.48]	-0.19 [-0.66, 0.28]
WL	3.63 (2.33)	3.61 (2.55)	-0.01 [-0.30, 0.28]	
Internet-related craving, relief, ICS (0-12)	*Interaction effect*: *b* = 0.56, *SE* = 0.57, *p* = .332			
PROTECTapp	2.36 (2.20)	2.39 (2.27)	0.02 [-0.35, 0.39]	-0.05 [-0.52, 0.42]
WL	3.04 (2.65)	2.52 (2.47)	-0.21 [-0.50, 0.08]	
Motivation to change, recognition, iSOCRATES (7-35)	*Interaction effect*: *b* = -0.39, *SE* = 0.78, *p* = .618			
PROTECTapp	22.54 (5.48)	21.64 (4.92)	-0.18 [-0.56, 0.19]	-0.07 [-0.54, 0.40]
WL	22.57 (5.75)	22.07 (6.68)	-0.07 [-0.36, 0.21]	
Motivation to change, ambivalence, iSOCRATES (4-20)	*Interaction effect*: *b* = -0.83, *SE* = 0.44, *p* = .066			
PROTECTapp	13.82 (1.98)	12.93 (2.48)	-0.36 [-0.74, 0.02]	-0.22 [-0.69, 0.25]
WL	13.61 (2.56)	13.54 (2.99)	-0.02 [-0.31, 0.27]	
Motivation to change, taking steps, iSOCRATES (8-40)	*Interaction effect*: *b* = 3.22, *SE* = 1.16, *p* = .008			
PROTECTapp	23.36 (5.77)	26.54 (5.90)	0.54 [0.14, 0.94]	0.43 [-0.05, 0.90]
WL	23.98 (5.63)	23.93 (6.17)	-0.01 [-0.30, 0.28]	
Internet gaming disorder symptoms, IGDS (0-9)	*Interaction effect*: *b* = -0.03, *SE* = 0.36, *p* = .942			
PROTECTapp	0.75 (1.27)	0.57 (1.07)	-0.17 [-0.54, 0.21]	-0.55 [-1.03, -0.08]
WL	1.61 (2.18)	1.46 (1.85)	-0.08 [-0.37, 0.21]	

(*To be continued*)

Table S3 (*continued*)

Measure (possible range)	Baseline	Post-intervention	Effect size *d* [95% CI]	
	M(*SD*)	*M*(*SD*)	Within (Pre/Post)	Between (Post)
Social media disorder symptoms, SMDS (0-9)	*Interaction effect*: *b* = -0.18, *SE* = 0.34, *p* = .599			
PROTECTapp	1.68 (1.33)	1.21 (1.50)	-0.31 [-0.69, 0.07]	-0.56 [-1.04, -0.09]
WL	2.37 (1.61)	2.09 (1.59)	-0.18 [-0.47, 0.11]	
Internalizing symptoms, PHQ-4 (0-12)	*Interaction effect*: *b* = 0.18, *SE* = 0.44, *p* = .682			
PROTECTapp	4.39 (2.56)	4.89 (3.12)	0.15 [-0.22, 0.52]	0.00 [-0.47, 0.47]
WL	4.59 (3.07)	4.87 (3.10)	0.09 [-0.20, 0.38]	
Social anxiety symptoms, mini-SPIN (0-12)	*Interaction effect*: *b* = -0.47, *SE* = 0.41, *p* = .251			
PROTECTapp	3.75 (2.66)	4.21 (2.92)	0.16 [-0.21, 0.53]	-0.12 [-0.59, 0.35]
WL	3.63 (3.03)	4.57 (3.06)	0.31 [0.01, 0.60]	
Well-being, WHO-5 (0-25)	*Interaction effect*: *b* = -0.44, *SE* = 0.82, *p* = .594			
PROTECTapp	12.46 (4.04)	11.89 (4.87)	-0.12 [-0.49, 0.25]	0.12 [-0.35, 0.59]
WL	11.46 (4.75)	11.33 (4.89)	-0.03 [-0.32, 0.26]	
Procrastination, PFS-4 (4-20)	*Interaction effect*: *b* = -0.06, *SE* = 0.54, *p* = .909			
PROTECTapp	11.79 (4.48)	12.07 (3.92)	0.07 [-0.30, 0.44]	-0.21 [-0.68, 0.26]
WL	12.67 (4.44)	13.02 (4.72)	0.15 [-0.22, 0.36]	
General self-efficacy, SWE (10-40)	*Interaction effect*: *b* = -1.07, *SE* = 0.69, *p* = .125			
PROTECTapp	27.68 (3.85)	27.00 (3.63)	-0.19 [-0.56, 0.19]	-0.30 [-0.77, 0.17]
WL	28.02 (5.09)	28.41 (5.24)	0.07 [-0.21, 0.36]	
Study-related self-efficacy, WIRKSTUD (7-28)	*Interaction effect*: *b* = -0.45, *SE* = 0.65, *p* = .494			
PROTECTapp	18.68 (3.82)	17.64 (3.92)	-0.26 [-0.64, 0.11]	-0.17 [-0.64, 0.30]
WL	18.91 (4.78)	18.33 (4.25)	-0.14 [-0.43, 0.15]	

Note. PROTECTapp, mobile-based approach-avoidance task training for problematic internet use (*n* = 28), WL, waitlist control group (*n* = 46). CIUS, Compulsive Internet Use Scale, ICS, Internet Craving Scale, iSOCRATES, Stages of Change Readiness and Treatment Eagerness Scale (adapted for internet use), IGDS, Internet Gaming Disorder Scale, SMDS, Social Media Disorder Scale, PHQ-4, Patient Health Questionnaire – ultra-brief screener, mini-SPIN, Mini Social Phobia Inventory, WHO-5, WHO-5 Well-being Index, PFS-4, Procrastination Questionnaire for Students, SWE, General Self-Efficacy Scale, WIRKSTUD, Study-specific Self-Efficacy Scale.

Table S4

Estimated means and observed SDs at follow-up assessments, model-based effects of time, and within-person effect sizes for primary and secondary outcomes in the intervention group (intention-to-treat sample)

Measure (possible range)	Model results			*M*(*SD*)	Effect size *d* [95% CI] (reference: pre)
	Estimate	*SE*	*p*		
Problematic internet use, CIUS (0-56)					
Baseline	-	-	-	32.04 (6.72)	-
Post-intervention	-6.24	1.30	< .001	25.80 (8.14)	-0.77 [-1.11, -0.42]
3-weeks follow-up	-6.87	1.35	< .001	25.17 (9.32)	-0.74 [-1.10, -0.37]
12-weeks follow-up	-8.67	1.38	< .001	23.38 (8.80)	-0.98 [-1.40, -0.57]
Internet-related craving, anticipation, ICS (0-12)					
Baseline	-	-	-	7.98 (2.21)	-
Post-intervention	-1.31	0.51	.013	6.67 (2.64)	-0.50 [-0.82, -0.18]
3-weeks follow-up	-1.57	0.48	.002	6.41 (2.73)	-0.57 [-0.92, -0.23]
12-weeks follow-up	-1.32	0.50	.013	6.66 (3.17)	-0.42 [-0.77, -0.06]
Internet-related craving, desire, ICS (0-12)					
Baseline	-	-	-	3.22 (2.40)	-
Post-intervention	0.07	0.39	.851	3.30 (2.24)	0.03 [-0.27, 0.34]
3-weeks follow-up	-0.50	0.35	.155	2.72 (2.87)	-0.17 [-0.50, 0.15]
12-weeks follow-up	-0.25	0.42	.561	2.98 (2.58)	-0.10 [-0.44, 0.25]
Internet-related craving, relief, ICS (0-12)					
Baseline	-	-	-	2.53 (2.61)	-
Post-intervention	-0.02	0.36	.946	2.51 (2.28)	-0.01 [-0.31, 0.29]
3-weeks follow-up	-0.29	0.36	.414	2.24 (2.52)	-0.12 [-0.44, 0.21]
12-weeks follow-up	-0.21	0.39	.602	2.33 (2.32)	-0.09 [-0.43, 0.25]
Motivation to change, recognition, iSOCRATES (7-35)					
Baseline	-	-	-	23.00 (5.73)	-
Post-intervention	-1.48	0.58	.015	21.52 (5.78)	-0.26 [-0.56, 0.05]
3-weeks follow-up	-2.67	0.84	.003	20.33 (7.02)	-0.38 [-0.71, -0.05]
12-weeks follow-up	-4.15	0.82	< .001	18.86 (5.95)	-0.70 [-1.08, -0.32]

(*To be continued*)

Table S4 (*continued*)

Measure (possible range)	Model results			*M*(*SD*)	Effect size *d* [95% CI] (reference: pre)
	Estimate	*SE*	*p*		
Motivation to change, ambivalence, iSOCRATES (4-20)					
Baseline	-	-	-	14.04 (2.18)	-
Post-intervention	-1.15	0.38	.004	12.90 (2.86)	-0.40 [-0.72, -0.09]
3-weeks follow-up	-1.60	0.43	< .001	12.45 (3.12)	-0.51 [-0.85, -0.17]
12-weeks follow-up	-2.47	0.41	< .001	11.58 (2.88)	-0.86 [-1.26, -0.46]
Motivation to change, taking steps, iSOCRATES (8-40)					
Baseline	-	-	-	23.18 (5.74)	-
Post-intervention	3.54	0.94	< .001	26.72 (6.36)	0.56 [0.23, 0.88]
3-weeks follow-up	1.85	1.22	.137	25.02 (7.97)	0.23 [-0.09, 0.56]
12-weeks follow-up	2.65	1.10	.021	25.82 (6.88)	0.38 [0.03, 0.74]
Internet gaming disorder symptoms, IGDS (0-9)					
Baseline	-	-	-	1.16 (1.64)	-
Post-intervention	-0.45	0.23	.061	0.71 (1.28)	-0.35 [-0.66, -0.04]
3-weeks follow-up	-0.66	0.29	.027	0.50 (1.15)	-0.58 [-0.92, -0.23]
12-weeks follow-up	-0.88	0.23	< .001	0.28 (0.64)	-1.38 [-1.86, -0.90]
Social media disorder symptoms, SMDS (0-9)					
Baseline	-	-	-	2.11 (1.91)	-
Post-intervention	-0.73	0.30	.019	1.38 (1.59)	-0.46 [-0.78, -0.14]
3-weeks follow-up	-0.86	0.27	.003	1.26 (1.59)	-0.54 [-0.88, -0.19]
12-weeks follow-up	-1.03	0.26	< .001	1.08 (1.60)	-0.65 [-1.02, -0.27]
Internalizing symptoms, PHQ-4 (0-12)					
Baseline	-	-	-	4.22 (2.50)	-
Post-intervention	0.64	0.35	.078	4.86 (3.22)	0.20 [-0.11, 0.50]
3-weeks follow-up	0.48	0.43	.274	4.70 (3.13)	0.15 [-0.17, 0.48]
12-weeks follow-up	0.20	0.58	.731	4.42 (3.47)	0.06 [-0.28, 0.40]
Social anxiety symptoms, mini-SPIN (0-12)					
Baseline	-	-	-	3.91 (2.70)	-
Post-intervention	0.48	0.32	.141	4.39 (2.98)	0.16 [-0.14, 0.47]
3-weeks follow-up	0.67	0.40	.096	4.59 (2.91)	0.23 [-0.09, 0.56]
12-weeks follow-up	-0.14	0.42	.748	3.77 (3.15)	-0.04 [-0.38, 0.30]

(*To be continued*)

Table S4 (*continued*)

Measure (possible range)	Model results			*M*(*SD*)	Effect size *d* [95% CI] (reference: pre)
	Estimate	*SE*	*p*		
Well-being, WHO-5 (0-25)					
Baseline	-	-	-	12.42 (3.88)	-
Post-intervention	-0.56	0.66	.403	11.87 (5.17)	-0.11 [-0.41, 0.20]
3-weeks follow-up	-0.54	0.69	.443	11.88 (5.33)	-0.10 [-0.42, 0.22]
12-weeks follow-up	-0.58	0.87	.508	11.84 (5.34)	-0.11 [-0.45, 0.23]
Procrastination, PFS-4 (4-20)					
Baseline	-	-	-	12.91 (4.40)	-
Post-intervention	-0.08	0.35	.829	12.84 (3.74)	-0.02 [-0.32, 0.28]
3-weeks follow-up	-0.05	0.45	.919	12.86 (4.73)	-0.01 [-0.33, 0.31]
12-weeks follow-up	0.41	0.56	.472	13.32 (4.92)	0.08 [-0.26, 0.42]
General self-efficacy, SWE (10-40)					
Baseline	-	-	-	28.11 (4.15)	-
Post-intervention	-0.82	0.45	.076	27.29 (3.99)	-0.21 [-0.51, 0.10]
3-weeks follow-up	-1.19	0.61	.058	26.92 (5.02)	-0.24 [-0.56, 0.09]
12-weeks follow-up	0.32	0.72	.657	28.43 (5.42)	0.06 [-0.28, 0.40]
Study-related self-efficacy, WIRKSTUD (7-28)					
Baseline	-	-	-	18.98 (3.51)	-
Post-intervention	-0.55	0.49	.272	18.43 (3.61)	-0.15 [-0.46, 0.15]
3-weeks follow-up	-0.21	0.44	.640	18.77 (3.65)	-0.06 [-0.38, 0.27]
12-weeks follow-up	-0.11	0.51	.837	18.87 (4.57)	-0.02 [-0.36, 0.32]

Note. CIUS, Compulsive Internet Use Scale, ICS, Internet Craving Scale, iSOCRATES, Stages of Change Readiness and Treatment Eagerness Scale (adapted for internet use), IGDS, Internet Gaming Disorder Scale, SMDS, Social Media Disorder Scale, PHQ-4, Patient Health Questionnaire – ultra-brief screener, mini-SPIN, Mini Social Phobia Inventory, WHO-5, WHO-5 Well-being Index, PFS-4, Procrastination Questionnaire for Students, SWE, General Self-Efficacy Scale, WIRKSTUD, Study-specific Self-Efficacy Scale.
